# Characterization of the Ornithine Hydroxylation Step in Albachelin Biosynthesis

**DOI:** 10.3390/molecules22101652

**Published:** 2017-10-01

**Authors:** Kendra Bufkin, Pablo Sobrado

**Affiliations:** 1 Department of Biochemistry, Virginia Tech, Blacksburg, VA 24061, USA; psobrado@vt.edu; 2 Center for Drug Discovery, Virginia Tech, Blacksburg, VA 24061, USA

**Keywords:** flavin, siderophores, *Amycolatopsis alba*, ornithine hydroxylase

## Abstract

*N*-Hydroxylating monooxygenases (NMOs) are involved in siderophore biosynthesis. Siderophores are high affinity iron chelators composed of catechol and hydroxamate functional groups that are synthesized and secreted by microorganisms and plants. Recently, a new siderophore named albachelin was isolated from a culture of *Amycolatopsis alba* growing under iron-limiting conditions. This work focuses on the expression, purification, and characterization of the NMO, abachelin monooxygenase (AMO) from *A. alba*. This enzyme was purified and characterized in its holo (FAD-bound) and apo (FAD-free) forms. The apo-AMO could be reconstituted by addition of free FAD. The two forms of AMO hydroxylate ornithine, while lysine increases oxidase activity but is not hydroxylated and display low affinity for NADPH.

## 1. Introduction

**Scheme 1:**
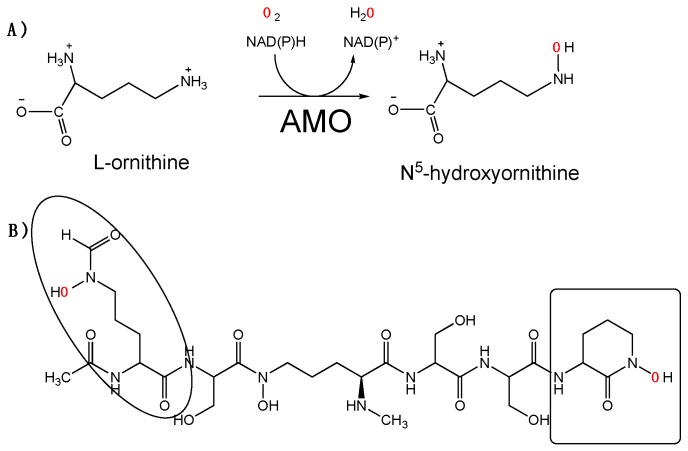
(**A**) Reaction catalyzed by abachelin monooxygenase (AMO). (**B**) Structure of albachelin. The hydroxylated ornithines produced by AMO are enclosed.

The actinomycetes *Amycolatopsis alba* produces the siderophore albachelin under iron-limiting conditions. Albachelin is a hydroxamate-containing siderophore composed of a linear peptide of 6 amino acids: *N*-α-acetyl-*N*-δ-hydroxy-*N*-δ-formylornithine, *N*-α-methyl-*N*-δ-hydroxyornithine, cyclic *N*-hydroxyornithine, and three molecules of serine (Scheme 1). Ornithine and its derivatives make up the backbone of the peptide and coordinate the iron. The operon that codes for the enzymes in albachelin biosynthesis includes a putative flavin-dependent ornithine monoxygenase [1]. We refer to this enzyme as albachelin monooxygenase (AMO) (Scheme 1). AMO is a member of the *N*-hydroxylating monooxygenase (NMO) family of enzymes. Most members of this enzyme family hydroxylate the N^5^ or N^6^ of an ornithine or lysine, respectively [2]. However, it has recently been shown that other NMOs catalyze *N*-atom hydroxylation of other natural products [3,4,5]. AMO is 30% identical to siderophore A (SidA) from *Aspergillus fumigatus*. SidA catalyzes the formation of *N*^5^-hydroxyornithine in the biosynthesis of hydroxamate-containing siderophores in *A. fumigatus* and is the best characterized NMO [6,7]. SidA activity is initiated by NADPH binding, followed by hydride transfer to the flavin. The complex, with reduced flavin and NADP^+^, reacts with oxygen to form the C4a-hydroperoxyflavin intermediate, which hydroxylates ornithine [8,9].

Here, we report the expression, isolation, and biochemical characterization of AMO. The enzyme was expressed and purified from *E. coli* using metal affinity chromatography. Depending on the imidazole concentration used, the recombinant enzyme was isolated either in the apo (FAD-free) or holo (FAD-bound) forms. Steady-state characterization shows that AMO can hydroxylate ornithine but not lysine; however, it displays very low selectivity for the reduced nicotinamide coenzyme. The biochemical and structural comparison to other NMOs is presented.

## 2. Results

### 2.1. Expression and Purification of AMO

**Figure 1:**
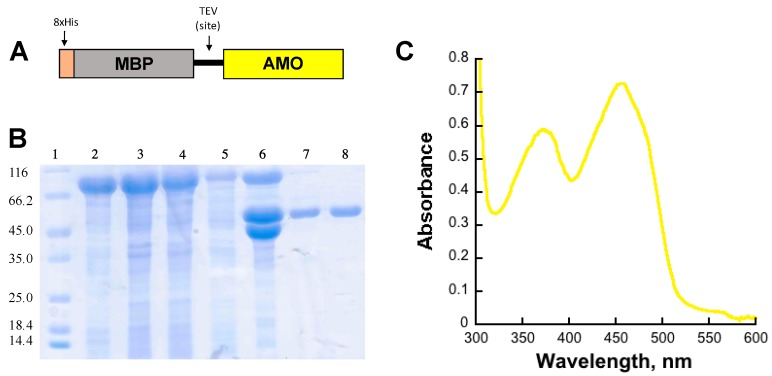
(**A**) Schematic of the fusion protein produced using the pVP56K vector. (**B**) SDS-PAGE gel summarizing the purification of AMO: lane 1: molecular weight markers, lane 2: cell pellet, lane 3: supernatant, lane 4: flow-through, lane 5: wash, lane 6: sample after cleavage with Tev, lane 7: holo-AMO, and lane 8: apo-AMO. (**C**). UV-visible spectrum of bound FAD in purified holo-AMO.

Recombinant AMO was expressed as a fusion to maltose binding protein (MBP) containing an N-terminus 8xHis tag (8xHis-MBP) in the pVP56K vector (Figure 1). The N-terminus fusion of AMO with an 8xHis-MBP tag was necessary to obtain a soluble protein. Additionally, the tag allowed the use of immobilized affinity chromatography (IMAC). AMO was cleaved from the 8xHis-MBP tag with tobacco etch virus (8xHis-TEV) protease and dialyzed to remove the imidazole used during elution. To isolate the cleaved AMO from the 8xHis-MBP and the 8xHis-TEV, the solution was loaded onto an IMAC column. The tag-free AMO had weak affinity to the IMAC column and was eluted with low concentrations of imidazole. At low imidazole concentrations (~1 mM), AMO with bound flavin (holo-AMO) eluted from the columns. A volume of 30 mM imidazole was used to elute flavin-free AMO (apo-AMO). The 8xHis-MBP and 8xHis-TEV were eluted with 300 mM imidazole. Both forms of AMO were purified at ~95% purity (Figure 1B). The UV-vis spectrum of recombinant holo-AMO shows absorbance peaks at 380 and 450 nm corresponding to a flavin cofactor (Figure 1C). The flavin extinction at 450 nm coefficient was calculated to be 12,000 M^−1^ cm^−1^ and the cofactor incorporation was 55–70%.

### 2.2. Steady-State Kinetics Varying Ornithine 

The apo-AMO was reconstituted (recon-AMO) by addition of free FAD in solution. The activity of holo- and recon- AMO was monitored by measuring the rate of oxygen consumption as a function of ornithine concentration while keeping NADPH constant at 5 mM. The *K*_M(Orn)_ and the *k*_cat_ values are ~3-fold and 1.7-fold higher, respectively, for holo-AMO as compared to recon-AMO (Table 1). However, the *k*_cat_/*K*_M(Orn)_ values are very similar between the apo- and recon-AMOs. The kinetic parameters with Lys were very similar to those obtained with Orn. Measuring the formation of hydroxylated ornithine, using the product formation assay, allowed calculation of a *k*_cat_ value for the holo-AMO, which was 20-fold higher than for recon-AMO. The *k*_cat_/*K*_M(Orn)_ value for holo-AMO is also ~20-fold higher because the *K*_M(Orn)_ values are almost identical for both apo- and recon-AMOs in the product formation assay. There was no product formation with Lys as the substrate.

### 2.3. Steady-State Kinetics Varying Reduced Nicotinamide Concentrations

The initial velocities as a function of NADPH or NADH at saturated ornithine concentration (10 mM) were determined by measuring oxygen consumption and product formation. With NADPH, the *k*_cat_ value calculated by measuring oxygen consumption was slightly lower for the holo-AMO and unchanged for the recon-AMO compared to the value obtained when ornithine was the substrate (Table 1 and Table 2). Using the same assay, holo-AMO showed lower *K*_M_ values for both the reduced nicotinamide enzyme compared to the recon-AMO. However, both AMOs show a slight preference for NADH, based on lower *K*_M_ values (Table 2). By measuring the formation of hydroxylated ornithine, the *k*_cat_ values with NADPH are 1.5- and 2.1-fold lower for the holo- and recon-AMO, respectively. Recon-AMO also showed a lower *K*_M_ value for NADH than for NADPH. Surprisingly, hydroxylation of ornithine was not detected with the holo-AMO when NADH was the reducing agent (Table 2).

### 2.4. Hydrogen Peroxide Formation

**Figure 2:**
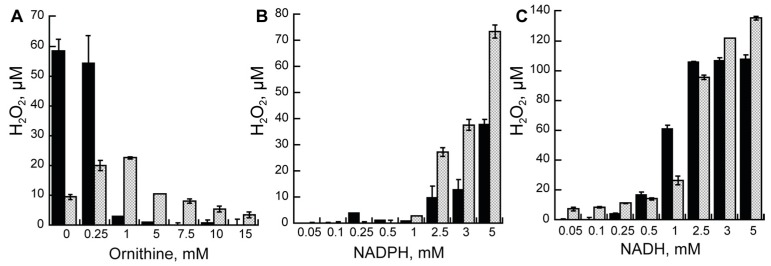
Hydrogen peroxide assay. (**A**) Hydrogen peroxide measured at increasing concentrations of Orn in the presence of 5 mM NADPH. Hydrogen peroxide at increasing concentrations of NADPH (**B**) or NADH (**C**) at saturating concentrations of Orn (10 mM). In all three experiments, recon-AMO is shown in black bars and holo-AMO is shown in dotted gray bars.

The difference in the *k*_cat_ values between the oxygen consumption assay and the product formation assay suggest that there is uncoupling in the reaction of AMO. This means that not all activated oxygen molecules form the C4a-hydroperoxyflavin, resulting in the hydroxylation of ornithine. Instead, hydrogen peroxide can be formed. Thus, we measured hydrogen peroxide formation in the reaction of AMO. Production of hydrogen peroxide as a function of Orn or reduced nicotinamide coenzyme concentration is shown in Figure 2. For both AMOs, there is a decrease in the amount of hydrogen peroxide as the concentration of Orn increases in the presence of NADPH.

At lower concentrations of Orn, the amount of hydrogen peroxide is greater for the holo-AMO but decreases to values close to zero at saturating concentrations. Even at a saturating concentration of ornithine, the amount of hydrogen peroxide decreased for the recon-AMO but not as much as for the holo-AMO. At saturating concentrations of ornithine (10 mM), low concentrations of NADPH did not lead to high levels of hydrogen peroxide. This is most likely due to the relatively low affinity of both AMOs for this coenzyme. At concentrations of NADPH higher than the *K*_M_ values, increased concentrations of hydrogen peroxide were detected. The values were higher for the recon-AMO. Hydrogen peroxide production with NADH was also lower at concentrations below the *K*_M_ values, and increased to much higher levels when the NADH concentration was higher. There were no major differences in hydrogen peroxide production between the AMOs with NADH.

## 3. Discussion

NMOs play a key role in the biosynthesis of hydroxamate containing siderophores [2,10]. These enzymes have been shown to be specific for the substrate that is hydroxylated. For instance, enzymes that hydroxylate ornithine are unable to hydroxylate lysine or do so with much lower efficiency [10,11]. Enzymes that are specific for ornithine have also been shown to display preference for NADPH. The best characterized ornithine monooxygenase is the enzyme from *A. fumigatus* known as SidA. SidA is produced and isolated with high levels of FAD bound (>90%) [6]. This enzyme has been shown to have higher affinity for NADPH (at least 100-fold higher K_D_) and the reaction nears 100% coupling. In contrast, the reaction is only 50% coupled with NADH [6,7,8,12,13]. PvdA is the ornithine monooxygenase from *P. aeruginosa*. When this enzyme is expressed in *E. coli*, the protein is isolated in the apo-form. Upon reconstitution with FAD, the protein is active and shows high affinity for NADPH and is highly coupled, but is unable to react with NADH [14,15]. Differences in affinity and coupling are also observed in lysine monoxygenases. The enzyme from *Mycobaterium smegmatis*, MbsG, has been shown to have low affinity for NAD(P)H (>1 mM) with an ~6-fold preference for NADH. Coupling is low but similar between the two coenzymes at only ~12–17% [16,17]. *Nocardia facinica* G, NbtG, is another lysine monooxygenase that has been well-characterized. NbtG displays a 4-fold preference to NADPH and is 70% coupled with NADPH as compared to only 50% with NADH [18,19]. Thus, the ornithine hydroxylases have been shown to have specificity for NADPH, while the lysine monooxygases show lower affinity with NADPH and are less specific, with some preferring NADH (MbsG) and others NADPH (NbtG). The expression and purification of recombinant AMO resulted in the isolation of two enzymes: apo- and holo-AMO. The recon-AMO was active after reconstitution with FAD. Both AMOs are specific for ornithine and display similar *K*_M_ values as reported for other ornithine monooxygenases [6,14]. Lysine binds and accelerates the rate of oxygen consumption; however, it does not get hydroxylated. Thus, lysine functions as an effector molecule, as has been shown in SidA and PvdA [6,14]. While *K*_M_ values for the reduced dinucleotides are usually in the low micromolar range for SidA or PvdA, they are significantly higher for AMO. The high *K*_M_ values are similar to those reported for MbsG and NbtG [16,17,18]. Furthermore, when measuring the oxygen consumption activity, both AMOs display preference for NADH. This feature has only been observed in the lysine monooxygense MbtG. Although based on *K*_M_ values there appears to be a preference for NADH, the holo-AMO is unable to hydroxylate ornithine with this nucleotide. This suggests that the C4a-hydroperoxyflavin is not stabilized with NADH, allowing hydroxylation, and decays to hydrogen peroxide. This is supported by the fact that hydrogen peroxide production is higher when NADH is used (Figure 2).

Several members of the NMO group of enzymes have been characterized and it is becoming clear that their biochemical properties are diverse. As described above, some ornithine hydroxylases are specific for NADPH and highly coupled. In contrast, many of the lysine monooxygenses are promiscuous for their utilization of reduced dinucleotides and are not efficiently coupled. Similarly, some proteins are isolated with bound flavin and others are in the apo-form. AMO is the first NMO that has been isolated in the holo- and apo-forms. This enzyme is specific for ornithine; however, the *K*_M_ values for the reduced coenzymes are high and not significantly different. This is in stark contrast to what is observed in SidA and PvdA, which show preference for NADPH. It seems that AMO behaves more like a lysine monooxygenase, which have low affinity for the reduced coenzymes and are not very specific. It is also worth mentioning that the recon-AMO, in general, has a lower activity than the holo-AMO in addition to lower coupling, suggesting that the reconstituted enzyme does not acquire an optimal conformation.

**Figure 3:**
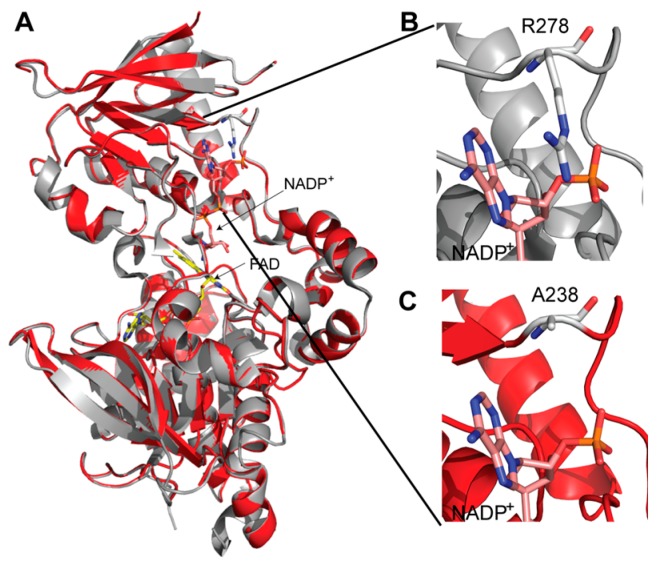
3-dimensional model of AMO. (**A**) The model of AMO (red) is overlaid on the structure of siderophore A (SidA) (PDB code 4B63, gray). FAD and NADP+ found in SidA are shown with sticks. (**B**) Interaction of R278 with the 2′-phosphate of NADP(H). This residue is responsible for the selectivity of NADPH over NADH. (**C**) At the position that corresponds to the R278, the model of AMO contains Ala238.

A three-dimensional model of AMO was created based on the structure of SidA. Using site-directed mutagenesis, it was shown that R278 is responsible for the specificity of SidA for NADPH over NADH. The structural model shows that AMO does not have a conserved Arg residue in this position; instead, it has an Ala (Figure 3). The lack of an Arg residue in addition to conformational changes that have been observed in the NAD(P)H binding domain might explain the differences in dinucleotide binding in AMO compared to SidA and PvdA. Further structural characterization of AMO will provide a better understanding of the mechanism of NMOs.

## 4. Materials and Methods

*E. coli* Turbo Bl21 (DE3) chemically competent cells were purchased from Invitrogen. Purification was performed on an AKTA Start FPLC (GE Healthcare, Chicago, IL, USA). l-Orn, l-Lys, buffers, salts, kanamycin, NADPH, NADH, 96 well-plates, and Pierce hydrogen peroxide detection kits were purchased from Thermo-Fisher Scientific. Oxygen consumption assays were done on a Hansatech Oxygraph (King’ Lynn, Norfolk, UK).

### AMO Expression and Purification

Turbo BL21 (DE3) *Escherichia coli* cells containing the pVP56K AMO plasmid were plated onto LB plates supplemented with 100 µg/mL kanamycin [20]. A single colony from the plates was used to inoculate two 50 mL LB medium flasks with 100 µg/mL of kanamycin. The culture was incubated overnight at 37 °C with continuous shaking at 250 rpm. Six 2.8 L Fernbach flasks, each containing 1 L of Terrific Broth (TB) auto-induction medium (phosphate buffer, succinic acid, MgSO_4_, and 30X media containing 15% (*w*/*v*) lactose, 24% (*v*/*v*) glycerol, and 0.45% (*w*/*v*) glucose) [21,22] were inoculated with 8 mL of overnight culture and kept at 37 °C with agitation at 250 rpm until the optical density at 600 nm (OD_600_) reached a value of approximately 5.0. The temperature was then reduced to 18 °C for 18 h. The cells were harvested by centrifugation at 4000× *g* for twenty minutes. The resulting cell paste (~65g) was stored at −80 °C until purification. Frozen cell pellets were resuspended in 200 mL of buffer A (25 mM HEPES buffer, pH 7.5, containing 300 mM NaCl and 25 mM Imidazole) with 1 mM phenylmethanesulfonylfluroide, 150 µM FAD, and 50 µg/mL of lysozyme and 25 µg/mL DNase and RNase each. The resuspended cells were mixed for 15 min at 4 °C then sonicated (Fischer Scientific Sonic Dismembrator Model 500) at 70% amplitude for fifteen minutes with a pulse of 5 s on and 10 s off in an ice bath. The lysate was centrifuged at 34,500 g for 1 h to remove cell debris. The resulting supernatant was then loaded onto a three-in-tandem 5 mL HisTrap FF crude column (GE Healthcare) previously pre-equilibrated with buffer A at a flow rate of 5 mL/min. After loading, the column was washed with buffer A, and bound recombinant AMO was eluted with 100% buffer B (25 mM HEPES buffer, pH 7.5, with 300 mM NaCl and 300 mM Imidazole) using 300 mM imidazole at a flow rate of 2 mL/min. After purification, fractions containing AMO were pooled and dialyzed overnight at 4 °C in buffer C (25 mM HEPES buffer, pH 7.5, containing 300 mM NaCl and 10% glycerol) containing 10 µg/mL TEV protease for MBP cleavage. The final imidazole concentration after dialyzing was less than 1 mM. The next day, the protein was removed from the dialysis bag and loaded at 2.5 mL/min onto 3 in tandem 5 mL His Trap FF crude columns equilibrated with buffer C. The holoenzyme form of AMO eluted at 1 mM imidazole, while the apo-AMO eluted at 30 mM imidazole. Fractions containing AMO (based on SDS-PAGE) were pooled, then diluted with storage buffer (25 mM HEPES pH 7.5, 150 mM NaCl, 10% Glycerol) and concentrated using a 30 kDa Amicon ^®^ Ultra Centrifugal filter membrane (Billerica, MA). Protein concentration was quantified using the Bradford Assay (MW 49 kDa) and the calculated extinction coefficient (ε450 = 12,000 M^−1^ cm^−1^) for holo-AMO. Aliquots were frozen in liquid nitrogen in approximately 20 µL beads prior to storage at −80 °C.

## 5. Determination of the Extinction Coefficient of Bound Flavin to Holo-AMO

The spectra of purified AMO in 100 mM sodium phosphate, pH 7.5, was recorded in a 1 cm path length quartz cuvette. After data collection, the sample was incubated at 95 °C for 10 min. The resulting solution was centrifuged for 5 min at 1500 rpm in a bench centrifuge and the supernatant removed to record the UV-Vis spectra of the liberated FAD. An extinction coefficient at 450 nm of 12,000 M^−1^ cm^−1^ was calculated for the amount of FAD bound to AMO using the extinction coefficient of 11,300 M^−1^ cm^−1^ for free FAD [23].

## 6. Oxygen Consumption Assay

Oxygen consumed by AMO was monitored using a Hansatech Oxygraph Plus System (Norfolk, VA, USA) in a 1 mL reaction cell. The standard assay buffer contains 100 mM sodium phosphate buffer, pH 7.5. For these assays, NADPH or NADH concentration was varied, Orn was held constant at 10 mM, and NAD(P)H was held constant at 5 mM for reactions where Orn or Lys concentration was varied. The assay was initialed with 2.5 µM (holo-AMO) or 5 µM (apo-AMO) AMO. For the apo-AMO, 15 µM of FAD was incorporated into the reaction for activation of the enzyme.

## 7. Product Formation Assay: Determination of *N*-hydroxylation

Using a variation of the Csaky iodine oxidation assay, the amount of hydroxylated product formed by AMO was determined [24,25,26]. The standard assay buffer was 100 mM sodium phosphate, pH 7.5. The reaction was started by the addition of enzyme (10 µM for apo and 5 µM for holo) to 10 mM Orn or 5 mM NADPH in a reaction volume of 100 µL (15 µM of FAD was incorporated into the reaction for apo-AMO). The reaction was incubated for 30 min before it was terminated by the addition of 50 µL of 0.2 N perchloric acid. After quenching, the mixture was centrifuged for 1 min at 13.2× *g* and then 50 µL of the supernatant was transferred to a clear, 96-well plate. The reaction mixture was neutralized by adding 47.5 μL of 10% (*w*/*v*) sodium acetate solution followed by 47.5 μL of 1% (*w*/*v*) sulfanilic acid in 25% (*v*/*v*) acetic acid. To each well, 19 μL of 0.1% (*w*/*v*) iodide in glacial acetic acid was added and the reaction was incubated at room temperature for 15 min. Then, 19 µL of 0.1 N sodium thiosulfate was used to remove the excess iodine from the solution. The color was developed by the addition of 19 µL of 0.6% (*w*/*v*) α-naphthylamine in 30% (*v*/*v*) acetic acid. The absorbance at 562 nm was measured after 15 min using a Spectra-Max M5 plate reader (Molecular Devices). The amount of hydroxylated product formed was determined using a hydroxylamine hydrochloride (0–300 µM) standard curve. All kinetic data were fitted using Kaleidagraph software (Synery, Reading, PA, USA). To obtain *k*_cat_ and *K*_M_ values, the initial rate data was fitted to the Michaelis-Menten equation.

## 8. Detection of Hydrogen Peroxide Formation

Hydrogen peroxide was quantified using the Pierce hydrogen peroxide detection kit as previously described [16,17]. The standard assay buffer was 100 mM sodium phosphate, pH 7.5. The amount of hydrogen peroxide formed was determined as a function of Orn and Lys (0–15 mM) and NADH and NADPH (0–6 mM) in 60 µL of reaction buffer. In assays where NADPH/NADH was varied, Orn was held constant at 10 mM and in reactions where NAD(P)H was held constant at 1 mM, Orn was varied. Reactions were initiated with 10 µM of AMO and allowed to proceed at 25 °C for 30 min with constant shaking at 750 rpm. 20 µL aliquots were then mixed with 200 µL working reagent composed of 100 mM sorbitol, 125 µM xylenol orange, and 250 µM ammonium ferrous(II) sulfate and 25 mM H_2_SO_4_ in water. The mixture was then incubated for 10 min at 25 °C and the absorbance was recorded at 595 nm. A hydrogen peroxide standard curve was used to quantify the amount of hydrogen peroxide produced by AMO.

## 9. Three-Dimensional Model

The program Pyre (http://www.sbg.bio.ic.ac.uk/phyre/) was used to create the threading-based 3-dimensional model of AMO [27]. Pymol was used to create Figure 3 [28].

## Figures and Tables

**Table 1:** Activity of AMO monitored by oxygen consumption and product formation varying ornithine concentrations. Conditions in 100 mM sodium phosphate buffer, pH 7.5, in 5 mM NADPH.

Assay	Parameter	Holo-AMO	Recon-AMO
Oxygraph	*k*_cat_ (s^−1^)	0.340 ± 0.030	0.200 ± 0.010
(Orn)	*K*_M(Orn) (_mM)	0.305 ± 0.108	0.107 ± 0.052
	*k*_cat_/*K*_M(Orn)_ (mM^−1^ s^−1^)	1.12 ± 0.410	1.70 ± 0.810
Oxygraph	*k*_cat_ (s^−1^)	0.340 ± 0.020	0.170 ± 0.010
(Lys)	*K*_M(Lys) (_mM)	0.560 ± 0.110	0.180 ± 0.070
	*k*_cat_/*K*_M(Lys)_ (mM^−1^ s^−1^)	0.630 ± 0.120	0.950 ± 0.040
Product formation	*k*_cat_ (s^−1^)	0.200 ± 0.006	0.010 ± 0.003
(Orn)	*K*_M(Orn)_ (mM)	0.234 ± 0.048	0.240 ± 0.035
	*k*_cat_/*K*_M(Orn)_ (mM^−1^ s^−1^)	0.860 ± 0.200	0.040 ± 0.010
	%Coupling (Orn)	59%	5%

**Table 2:** Activity of AMO monitored by oxygen consumption and product formation with varying concentrations of reduced nicotinamide coenzymes. * Conditions are 100 mM sodium phosphate buffer, pH 7.5, in 10 mM Orn.

Assay *	Parameters	Holo-AMO		Recon-AMO	
		NADPH	NADH	NADPH	NADH
**Oxygraph**	*k*_cat_, (s^−1^)	0.25 ± 0.01	0.15 ± 0.01	0.17 ± 0.01	0.18 ± 0.01
	*K*_M(NAD(P)H_, (mM)	1.40 ± 0.16	0.32 ± 0.10	2.70 ± 0.35	1.60 ± 0.20
	*k*_cat_/*K*_M(NAD(P)H_, (mM^−1^ s^−1^)	0.160 ± 0.015	0.50 ± 10	0.060 ± 0.005	0.11 ± 0.01
**Product formation**	*k*_cat_, (s^−1^)	0.16 ± 0.05	N/A	0.08 ± 0.03	0.040 ± 0.010
	*K*_M(NAD(P)H_, (mM)	0.60 ± 0.20	N/A	2.60 ± 2.0	0.60 ± 0.30
	*k*_cat_/*K*_M(NAD(P)H_, (mM^−1^ s^−1^)	0.260 ± 0.020	N/A	0.03 ± 0.01	0.08 ± 0.02
	%Coupling	64%		47%	22%
